# Revealing bacteria-phage interactions in human microbiome through the CRISPR-Cas immune systems

**DOI:** 10.3389/fcimb.2022.933516

**Published:** 2022-09-28

**Authors:** Mahsa Monshizadeh, Sara Zomorodi, Kate Mortensen, Yuzhen Ye

**Affiliations:** Indiana University, Bloomington, IN, United States

**Keywords:** bacteria-phage interaction, mobile genetic elements (MGE), CRISPR-Cas systems, spacer, wound microbiome, pangenome

## Abstract

The human gut microbiome is composed of a diverse consortium of microorganisms. Relatively little is known about the diversity of the bacteriophage population and their interactions with microbial organisms in the human microbiome. Due to the persistent rivalry between microbial organisms (hosts) and phages (invaders), genetic traces of phages are found in the hosts’ CRISPR-Cas adaptive immune system. Mobile genetic elements (MGEs) found in bacteria include genetic material from phage and plasmids, often resultant from invasion events. We developed a computational pipeline (BacMGEnet), which can be used for inference and exploratory analysis of putative interactions between microbial organisms and MGEs (phages and plasmids) and their interaction network. Given a collection of genomes as the input, BacMGEnet utilizes computational tools we have previously developed to characterize CRISPR-Cas systems in the genomes, which are then used to identify putative invaders from publicly available collections of phage/prophage sequences. In addition, BacMGEnet uses a greedy algorithm to summarize identified putative interactions to produce a bacteria-MGE network in a standard network format. Inferred networks can be utilized to assist further examination of the putative interactions and for discovery of interaction patterns. Here we apply the BacMGEnet pipeline to a few collections of genomic/metagenomic datasets to demonstrate its utilities. BacMGEnet revealed a complex interaction network of the *Phocaeicola vulgatus* pangenome with its phage invaders, and the modularity analysis of the resulted network suggested differential activities of the different *P. vulgatus*’ CRISPR-Cas systems (Type I-C and Type II-C) against some phages. Analysis of the phage-bacteria interaction network of human gut microbiome revealed a mixture of phages with a broad host range (resulting in large modules with many bacteria and phages), and phages with narrow host range. We also showed that BacMGEnet can be used to infer phages that invade bacteria and their interactions in wound microbiome. We anticipate that BacMGEnet will become an important tool for studying the interactions between bacteria and their invaders for microbiome research.

## Introduction

Bacteriophages, or phages, are viruses that invade bacterial and archaeal species. Bacteria–phage coevolution functions as a driver of ecological and evolutionary processes in microbial communities (Koskella and Brockhurst, 2014). Due to the size difference between viral and bacterial genetic material, metagenomic sequencing projects typically focus on either bacteria or viruses (including phages), but not both. Special treatments such as size fractionation prior to DNA extraction can be used to reduce sources of nonviral DNA in viromes, enabling the recovery of richer viral populations relative to total metagenomes (Santos-Medellin et al., 2021). Advances in metagenomic sequencing and computational tool development have enabled the accumulation of a large number of metagenomic data sets, which were used to derive metagenome-assembled genomes (MAGs) (Almeida et al., 2021) and putative phages (Camarillo-Guerrero et al., 2019). Still, existing efforts focus on either side (bacteria or phages), but not both. As an example, a 2019 study (Hendriksen et al., 2019) found a systematic regional difference in the bacterial population and antimicrobial resistance gene (ARG) in global urban sewage, and only a more recent study (Strange et al., 2021) reanalyzed the published datasets to identify phages associated with bacteria and to explore their potential role in ARG dissemination.

CRISPR-Cas systems are highly prevalent in microbial genomes and can be grouped into two main classes, each of which contain multiple types (Barrangou et al., 2007; Levy et al., 2015; Koonin et al., 2017; Shmakov et al., 2017; Shmakov et al., 2018). Class 1 CRISPR-Cas Systems includes Types I, III and IV and use a complex of Cas proteins to degrade foreign nucleic acids. Class 2 CRISPR-Cas Systems include Types II, V, and VI and use a single, large Cas protein for the same purpose (Type II, V and VI use Cas9, Cas12 and Cas13, respectively) (Makarova et al., 2017). CRISPR arrays are comprised of short DNA segments, known as spacers, and these provide a cornerstone to CRISPR-Cas derived adaptive immunity. Spacers retain the memory of past immunological encounters, and are primarily acquired as a result of Cas protein complex mediated acquisition (Koonin et al., 2017). Newly acquired spacers are typically integrated towards the leader ends of arrays (Weinberger et al., 2012; McGinn and Marraffini, 2019). Estimates of species carrying CRISPR-Cas systems vary, and cautious interpretation of these estimates with close attention to context is advised. As an example, our recent analyses of the CRISPR-Cas systems in healthcare related pathogens showed that species who are normally void of CRISPR-Cas systems, such as, *Staphylococcus aureus*, contained CRISPR-Cas systems in a small fraction of isolates (0.55% of 12,212 isolates) (Mortensen et al., 2021). While this may seem like a small number, approximately 67 *S. aureus* isolates contained CRISPR-Cas systems, demonstrating the importance of context when analyzing CRISPR-Cas systems.

CRISPR spacers are genetic traces of invaders that are stored in host genomes. As such, CRISPR spacers have proven useful in phage-host prediction, either alone or in combination with other signals. This is demonstrated in a recently published tool CRISPROpenDB that utilizes CRISPR spacers, predicted from NCBI microbial genomes, in predicting phage membership to hosts (Dion et al., 2021). Results from this study were promising, achieving 49% recall and 69% precision (Dion et al., 2021). This approach is different from earlier computational approaches for phage host predictions, which mostly rely on sequence homology. Sequence homology-based approaches in phage host predictions aim to find similar phages to the phage of interest, or matches between the phage of interest and a genome integrated prophage in the bacterial host (Edwards et al., 2016). HostPhinder (Villarroel et al., 2016) is an exemplar based on finding similar phages for prediction of phage host. Other phage-host signals that have been exploited for phage host prediction include co-occurrence of phages and hosts across environments and correlations in nucleotide usage profiles (see this paper (Edwards et al., 2016) for a comparison of the strengths of the different phage-host signals for prediction).

Here we address a relevant but distinct computational problem, which is to infer the phages (and plasmids) that are likely to be the invaders of a collection of genomes and to infer the interaction network between the phages (invaders) and genomes (hosts). CRISPROpenDB takes phage sequences as input, and attempts to predict putative bacterial hosts based on pre-calculated CRISPR spacers from the bacterial genomes. By contrast, our pipeline BacMGENet takes a collection of genomes, the host(s), as the input and attempts to annotate the CRISPR-Cas systems in those genomes using our previously published tool CRISPRone (Zhang and Ye, 2017), and search identified CRISPR spacers against publicly available phage/plasmid sequences we collected to identify putative invaders that were defended against by the CRISPR-Cas systems. The collection of genomes can be a collection of different isolates of the same species (e.g., pangenome), or all the genomes found in a microbiome (e.g., wound microbiome and gut microbiome). We attempt to obtain a network of bacteria-phage interaction because there are phages that have a broad range of hosts. The inferred network will allow us to provide a more complete view of the microbiome in context of both the microbial species and phages, even for the studies that only focus on the bacteria, by taking advantage of the publicly available large collections of phage/plasmid sequences derived from metagenome sequencing studies. Interaction networks are also useful in studying the interaction patterns driven by the ongoing arms-race between bacteria and phages.

## Materials and Methods

**Figure 1:**
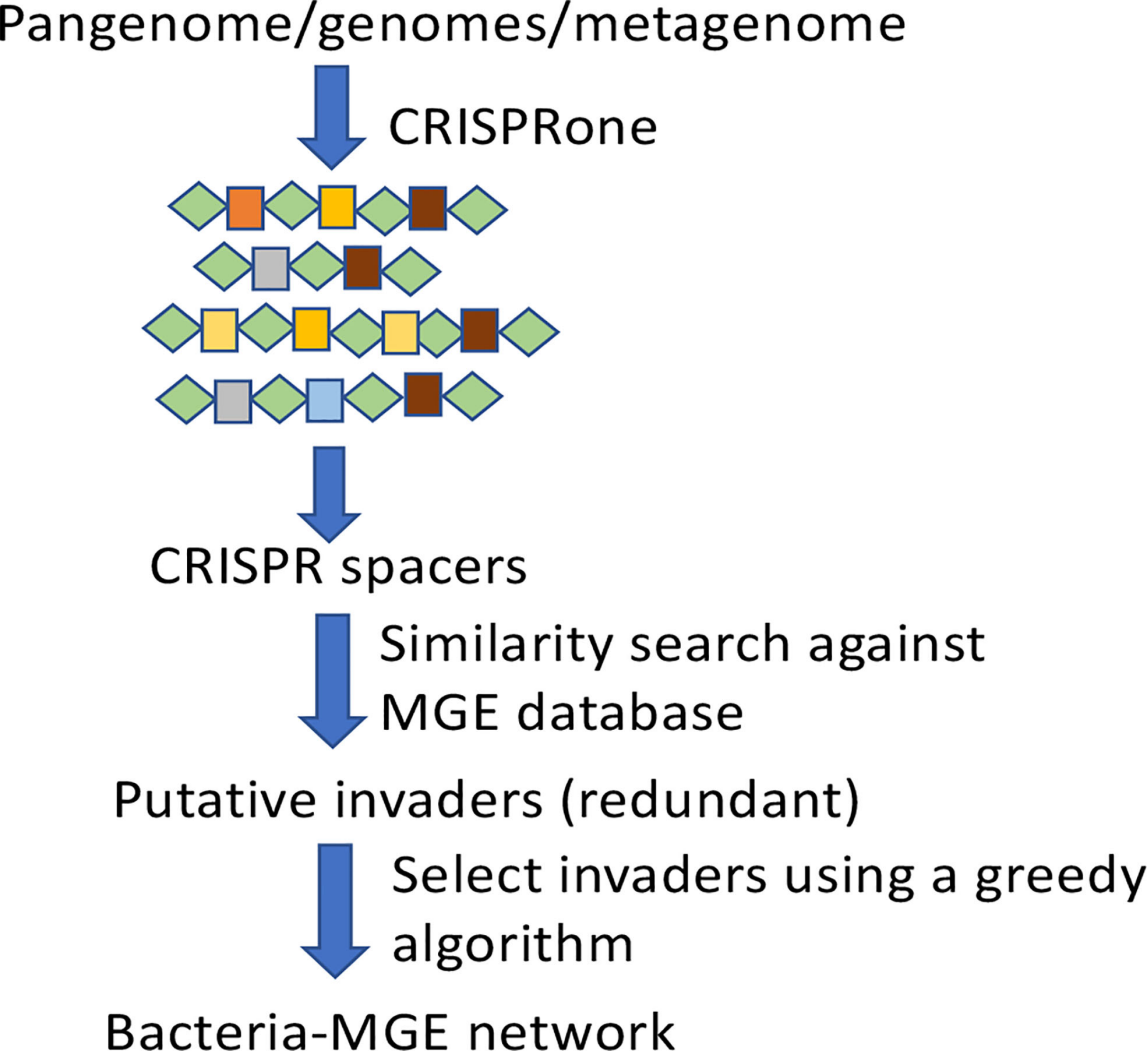
A schematic diagram of the BacMGEnet pipeline.

The BacMGEnet pipeline (**Figure 1**) reveals potential interactions between input microbial species (as a collection of genomes or in a metagenome) and their putative MGEs, and summarizes the putative interactions in a network. It utilizes computational tools that we have previously developed for characterizing CRISPR-Cas systems in genomes/metagenomes, and the large collections of MGE sequences that are currently available to identify potential MGEs with their traces found in predicted CRISPR-Cas systems in the bacterial genomes.

### Collection of the MGE datasets

We gathered a collection of mobile genetic element (MGE) databases, and collectively refer to these databases as the ‘MGE database’ for simplicity. The MGE database includes phage and plasmid sequences. The phage sequences were collected from the Gut Phage Database (Camarillo-Guerrero et al., 2019) (GPD), MicrobeVersusPhage (Gao et al., 2018) (MVP) database, the reference viral database (Goodacre et al., 2018) (RVDB), and mMGE (a database for human metagenomic extrachromosomal mobile genetic elements) (Lai et al., 2021). The GPD, MVP, RVDB and mMGE collections we used contain 142809, 32825, 825901, and 421635 entries, respectively. The plasmid sequences were collected from the Comprehensive and Complete Plasmid Database (Douarre et al., 2020) (COMPASS), and PLSDB (Galata et al., 2019). The phage and plasmid databases included sequences from the NCBI reference database, NCBI nucleotide database, prophages identified in prokaryotic genomes and MGEs identified from metagenomic assemblies. We made available the MGE database we collected for users to download and use.

### Identification of the CRISPR arrays

Our pipeline provides two different methods of characterizing CRISPR arrays in genomes or metagenome assemblies, from which spacers can be extracted for predicting host-MGE interactions. The first approach utilizes CRISPRone, a pipeline we previously developed for annotating CRISPR-Cas systems including CRISPR arrays and associated *cas* genes (Zhang and Ye, 2017). Identification of CRISPR arrays can be challenged by repetitive sequences that mimic CRISPR array structures (CRISPR artifacts). CRISPRone uses an ensemble method to remove potential false-positives such as tandem repeats and STAR-like sequence (Zhang and Ye, 2017). The second approach (also available in CRISPRone) uses known CRISPR repeats to guide the discovery of CRISPR arrays such that only CRISPR arrays containing identical, or very similar, repeats are included for analyses. The use of repeat guides, as in the latter approach, is advantageous because it reduces the possibility of including unwanted CRISPR artifacts and enforces precise boundaries around spacers. This is in contrast to *de novo* prediction where the detection of CRISPR arrays is purely based on the repeat-spacer repetitive structure. In cases where repeats are known, or users are interested in specific repeats associated with CRISPR-Cas systems, the guided prediction approach can be useful. We demonstrate the use of both approaches in this study.

### Identification of the interaction between microbial organisms and phages using the CRISPR arrays

Once CRISPR arrays are characterized in microbial genomes, spacers are extracted from identified CRISPR arrays and used for identification of invaders containing segments that match the spacers (i.e., protospacers). Unique spacers (100% nonredundant by CD-HIT-EST (Li and Godzik, 2006)) are queried against the MGE database using BLASTN (Camacho et al., 2009) to search for putative invaders that were targeted by the hosts containing the CRISPRs. All unique spacers are used in this analysis to increase the search sensitivity. Results are filtered to retain hits with a greater than 90% sequence identity, query coverage per hsp greater than 80%, and an e-value of less than 0.001. These parameters were used to ensure good matches between potential protospacers and spacers, and at the same time to allow a small number of mismatches between them caused by mutations or sequencing errors. Similar practice was used in previous work including our own (Zhang et al., 2013; Edwards et al., 2016; Dion et al., 2021).

A greedy algorithm is applied to select the minimum number of MGEs that collectively contain all protospacers matching the spacers. This step is necessary as the redundancy of the sequences in the MGE database is high, and including all MGEs that contain matching protospacers will make the network unnecessarily complex. The greedy algorithm works as follows. The MGEs are first sorted in descending according to the number of protospacers they contain. The MGE that contains the largest number of protospacers is selected (all the protospacers that this MGE contains are then considered to be covered or explained). The remaining MGEs are re-sorted according to the protospacers that they contain and are not yet covered by previously selected MGEs. The MGE containing the largest number of the protospacers is then selected. This process is repeated until all protospacers are covered by selected MGEs. Similarly, the greedy algorithm is applied to select the minimum number of hosts that contained all identified spacers and only included them in the network. Selected MGEs and hosts are then used for building spacer-MGE and host-MGE networks. In the spacer-MGE network, spacer sequence clusters (called spacers for simplicity) and MGEs are represented as nodes and an edge is added between a spacer node and MGE node if the MGE contains a segment that matches the spacer (i.e., protospacer). In the host-MGE network, an edge is added to a host and a MGE if the host and MGE pair contain at least one matching protospacer and spacer.

The spacer-MGE and host-MGE network can be of different uses; for example, the spacer-MGE network can be used to compare the involvement of different types of CRISPR-Cas systems in the interaction of bacteria and phages, and host-MGE network can be used for comparison of the interaction between phages and different bacteria. We used NetworkX (https://networkx.org/) to analyze all the networks that we inferred in this study, e.g., to compute connected components. All visualizations and manual inspection of the networks are performed using Cytoscape (Shannon et al., 2003).

### *Phocaeicola vulgatus* genomes

*P. vulgatus* is one of the commonly found bacterial species in human microbiome. In previous work, we showed that *P. vulgatus* is one of the generalists that are found in gut microbiomes of healthy individuals and individuals with diseases using metaproteomics datasets (Stamboulian et al., 2022). This served as motivation to include *P. vulgatus* in our current study. We downloaded *P. vulgatus* genomes from the NCBI ftp site (with the most release on March 17, 2022). In total there are 403 genomes, with 7 complete and 396 draft genomes.

### Human gut genomes

We used the human gut metagenomic-assembled genomes (MAGs) derived from 12 fecal samples (Jin et al., 2022b) for our gut bacteria-phage interaction prediction. This data is one of the most recent collections of human gut MAGs and it was shown that the use of a HiSeq-PacBio hybrid, ultra-deep metagenomic sequencing approach helped improve the sequencing coverage of the low-abundance subpopulation in the gut microbiome (Jin et al., 2022b). We downloaded a total of 472 MAGs from this website (Jin et al., 2022a).

### Wound microbiome and data processing

We also applied our tools to analyze wound microbiomes. We downloaded 196 metagenomic shotgun sequencing datasets of diabetic foot ulcer microbiome (Kalan et al., 2019) from the NCBI short reads archive (BioProject Accession PRJNA506988). Since some of these wound microbiome datasets have a large fraction of human reads (Kalan et al., 2019), we first applied Kraken2 and Bracken (Lu and Salzberg, 2020) to quantify the taxonomic composition (and also bacterial reads) for these datasets. We then selected the five wound microbiome datasets that contain the most non-human reads: SRR8247654 (referred as w1), SRR8247673 (w2), SRR8247619 (w3), SRR8247751 (w4) and SRR8247633 (w5) for analysis in this paper.

We assembled the five wound microbiome datasets. The sequencing data was first trimmed with Trimmomatic version 0.39 (Bolger et al., 2014). Next, the output of the trimming tool were mapped to the human reference genome assembly or GRCH38 using bowtie2 (Langmead and Salzberg, 2012) to remove human reads. The non-human reads were assembled using SPAdes version 3.15.4 using the –meta flag (Nurk et al., 2017).

### Availability of the pipeline

We made available our computational pipeline for bacteria-MGE interaction network inference given characterized CRISPR arrays (called mge_net), as well as a pipeline for characterizing CRISPR arrays redin genomes/metagenomes (called crispr ann) as a GitHub repository (called BacMGEnet) at https://github.com/mgtools/.BacMGEneta as open source codes. In addition to using the MGE database we provide, users can make their own customized database for discovery of MGEs that interact with the bacteria they are interested in. Our pipeline outputs annotations (if available) and the fasta sequences of identified phages, and their interactions with bacterial hosts in a standard network format. Users can use the sequences and apply other phage annotation tools such as PhaGCN (Shang et al., 2021) to assign the taxonomic groups such as ICTV families (Lefkowitz et al., 2018). Results of the examples reported in this paper are also available at the same repository.,

## Results

### Using *P. vulgatus* pangenome to identify its invaders

**Figure 2:**
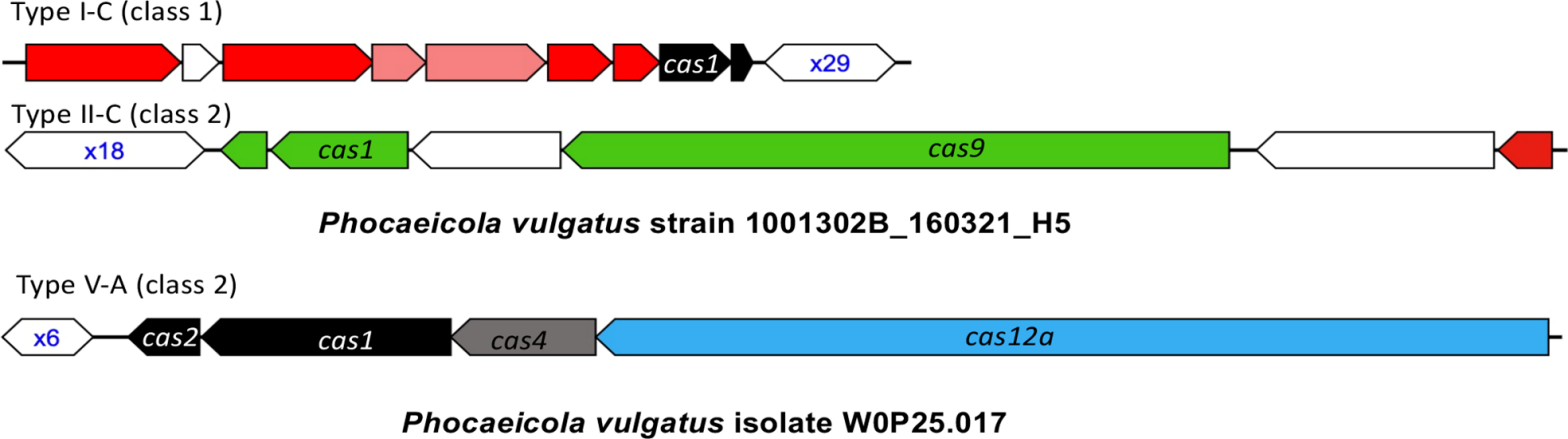
The CRISPR-Cas systems found in *P. vulgatus* pangenome. The genes and CRISPR arrays are shown as arrows and hexagons in the plots, respectively, with CRISPR arrays labelled by their number of repeats in blue text. The *cas* genes associated with different CRISPR-Cas types are shown in different colors, with a few important genes labelled by their gene names including *cas9* in Type II-C and *cas12a* in Type V CRISPR-Cas systems.

The *P. vulgatus* pangenome contains three types of CRISPR-Cas systems, among which Type V is the rarest, found in only one of the 403 P*. vulgatus* isolates (isolate W0P25.017). The other two types of CRISPR-Cas systems are more prevalent, with Type I-C CRISPR-Cas systems found in 169 (42%) genomes, and Type II-C CRISPR-Cas systems in 79 (20%) genomes. In total, about half of the isolates (213, 53%) contain at least one type of CRISPR-Cas systems in their genomes. **Figure 2** shows the representative structures of these three CRISPR-Cas systems found in the pangenome. A total of 1532 spacers were identified from the 213 P*. vulgatus* genomes containing CRISPR-Cas systems, among which 1190 (78%) spacers had hits in the MGE database, leading to identification of a total of 277 MGEs (260 phages and 17 plasmids)—these MGEs collectively contain all the protospacers that match the identified spacers. Among the 277 phages, 73 (26%) were from the GPD collection and have host predictions. Based on the pangenome-level analysis, we predict *P. vulgatus* as one of the putative hosts of all the remaining 204 phages.

**Figure 3:**
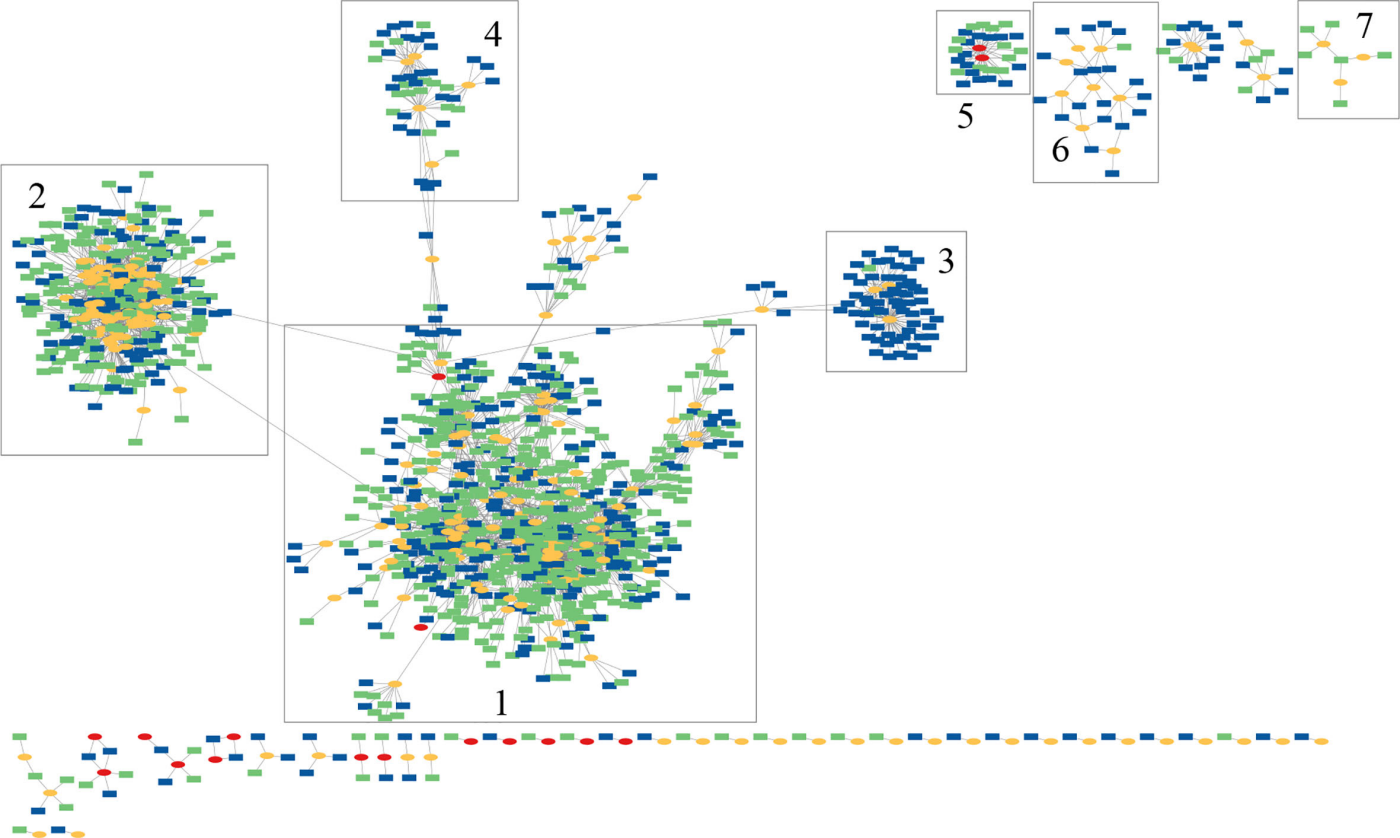
Spacer-host network inferred for *P. vulgatus* pangenome and its putative invaders. In this figure, CRISPR spacers found in *P. vulgatus* isolates/genomes are shown in green or blue rectangles (Type I-C spacers in green and Type II-C spacers in blue). Phages and plasmids are shown as yellow and red ovals, respectively.

A network of the spacers and MGEs was created (see **Figure 3**), in which the spacers and MGEs are the nodes and there is an edge between a spacer and a MGE if the MGE contains a segment matching the spacer. The network contains two large, highly connected modules (module 1 and 2 highlighted in **Figure 3**), each containing many Type I-C and Type II-C spacers, indicating interactions between different *P. vulgatus* isolates and phages (and their variants). Examination of the network also reveals a few modules (which are smaller) that mostly only contain spacers associated with one CRISPR-Cas system type; for example, module 3 and module 6 mainly contain Type II-C spacers, whereas module 7 only contains Type I-C spacers, suggesting differential activities of the different types of CRISPR-Cas systems against some of the invaders. Specifically, module 3 contains 4 phages and 61 spacers, among which 59 are Type II-C spacers and only 2 are Type I-C spacers. Although Type V-A CRISPR-Cas system was found in one of the *P. vulgatus* genomes, no protospacers were found in the MGE database that match the CRISPR spacers in this Type V-A CRISPR-Cas system and therefore Type V-A spacers are absent from this network.

### Phages with a broad spectrum of host ranges in human microbiome

Application of our pipeline to the collection of 472 human gut MAGs resulted in a collection of 8488 unique CRISPR spacers, among which 3812 (45%) found matching protospacers in the MGE database. Using these spacers as tags revealed a complex interaction network between gut microbial organisms and their putative invaders. The network contains 1871 nodes, including 237 nodes of microbial organisms (i.e., 54% of the MAGs are included in the network), and the remaining 1634 MGE nodes. Majority of the MGEs(1607) are phages, and only 27 are plasmids. Among the bacterial MAGs with their invaders identified through the CRISPR-Cas systems, 236 are bacteria, and only one archaeon (MAG ID: Y7.M001, a *Methanobrevibacter_A smithii*). Although rare, archaea are found to be important residents in human gut (Gaci et al., 2014).

**Figure 4:**
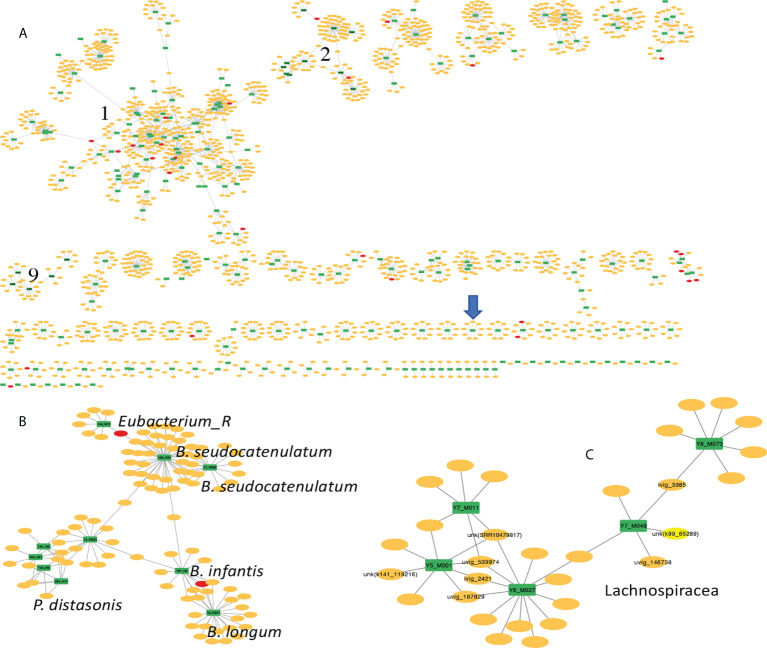
Human gut bacteria-MGEs interaction network. In this figure, bacteria hosts are shown in green rectangles, phages and plasmids are shown as yellow and red ovals, respectively. **(A)** is the global view of the network composed of 96 connected components. Three modules are highlighted with numbers; component 1 is the largest connected module containing many MAGs and putative MGEs. The blue arrow highlights the small component representing the interactions between the only archaeon found in this collection of human gut MAGs and nine putative MGEs. **(B)** and **(C)** show zoomed-in views of component 2 and 9, respectively. There are five *P. distasonis* bacteria in **(B)** and all bacteria host in component 9 (green nodes) are Lachnospiracea **(C)**.

Analysis of the bacteria-MGE network reveals some interesting patterns. The network contains a total of 96 connected components (see **Figure 4**). All components, except the second largest component (see **Figures 4A, B**) and the seventh largest component, each contain only bacteria from a phylum (if we don’t distinguish Firmicutes A (Almeida et al., 2021) and Firmicutes). Notably, the biggest component has 575 nodes including 73 bacteria all belonging to Firmicutes (but these bacteria represent at least 32 species including *Tyzzerella nexilis*, *Fusicatenibacter saccharivorans* and *Faecalicatena faecis*).

The second largest component (see **Figure 4B**) contains 110 nodes with 10 bacteria nodes and 100 MGE nodes; the 10 bacteria belong to three different phyla, including five Bacteroidota (*Parabacteroides distasonis*), four Actinobacteria (one *Bifidobacterium infantis*, two *Bifidobacterium pseudocatenulatum*, and one *Bifidobacterium longum*) and one Firmicutes A (*Eubacterium_R* sp000436835). The other component that contains bacteria from different phyla is the seventh largest component, which contains five Firmicutes bacteria and one Bacteroidota. All the results show that although there are specific interactions between certain phages and certain bacteria (such as the many small components with bacteria largely belonging to a specific clade), there are cases with interconnected interactions between phages and bacteria even from different phyla.

**Figure 4C** shows the 9th largest component representing interactions between five bacteria of family Lachnospiraceae (Firmicutes_A) and their putative phage invaders. The MAGs of the five bacteria are Y7_M011, Y5_M001, Y6_M027, Y7_M048, and Y8_M073 with the latter three sharing 0.95 ANI with UHGG genomes (Almeida et al., 2021) GUT_GENOME095973, GUT_GENOME000706, and GUT_GENOME000818, respectively. No finer taxonomic assignment was available for these five Lachnospiraceae. Y7_M011 and Y5_M001 are among the 24 new MAGs that were not found in any public genome database, thanks to the application of hybrid, ultra-deep metagenomic sequencing according to this paper (Jin et al., 2022b). This component represents a case where phages interact with a specific clade of bacteria in this case Lachnospiraceae. Among the 25 putative invaders (all are phages) that have protospacers matching the CRISPR spacers in these Lachnospiraceae genomes, ivig_2421 has protospacers matching Y5_M001 and Y6_M027, and uvig_539974 has protospacers matching CRISPR spacers in Y5_M001, Y6_M027 and Y7_M048 (these phages are highlighted with labels in **Figures 4**). According to GPD annotation, these two phages' bacteria hosts are Dorea scindens (Lachnospiraceae). Our component-based network analysis reveals phage-bacteria interaction that is consistent with the GPD annotation, and suggests potential bacterial host for phages with unknown host such as unk (SRR10479817P) in **Figure 4C**

Above we showcased a few representative components found in the human gut bacteria-MGE network. We note that the network (in the gml format) can be used by users programatically (e.g., by using functions available in NetworkX red(https://networkx.org/)) or visually (e.g., in Cytoscape (Shannon et al., 2003)). As an example to demonstrate that the network can be used to search for specific genome and its invaders, searching for Y7.M001 in Cytoscape visualization of the human gut bacteria-MGE newwork resulted in a small module, highlighted in **Figure 4A**, revealing the interactions between the only archaeon found in this collection of human gut MAGs with nine putative MGEs.

### Bacteria-phage interaction in wound microbiome

**Figure 5:**
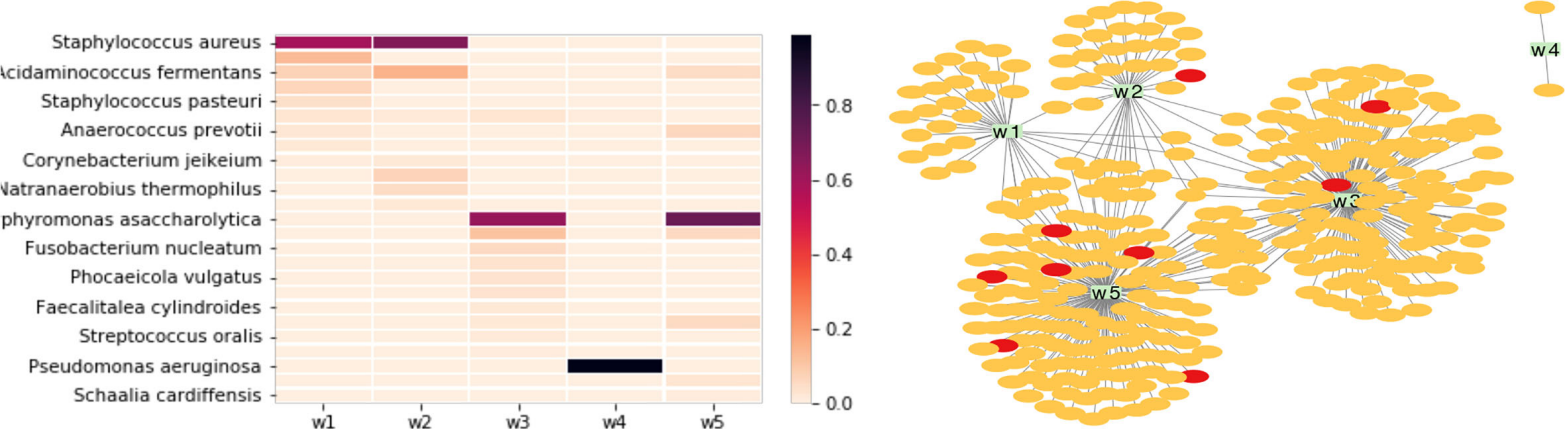
Comparison of taxonomic composition and phage-bacteria interaction in five wound microbiomes. The wound microbiome datasets are: w1 (SRA accession number: SRR8247654), w2 (SRR8247673), w3 (SRR8247619), w4 (SRR8247751), and w5 (SRR8247633). Left: the heatmap of the relative abundances of differnet bacterial species found in the microbiomes. Right: network of microbiome and its MGEs; in this figure, the yellow and red nodes represent different phages and plasmids that contain protospacers matching spacers found in the microbiomes, and each green rectangle represents a microbiome (w1-w5).

We first analyzed the taxonomic composition of the chosen wound microbiome datasets (w1-w5). **Figure 5** (left) shows the heatmap of the relative abundances of detected bacterial species in the five microbiome datasets (only species that were found in at least one of the samples with at least 1% relative abundance were included): *Staphylococcus aureus* is the dominant species in w1 and w2, *Porphyromonas asaccharolytica* is the dominant species in wound microbiome w3 and w5, and *Pseudomonas aeruginosa* is the dominant species in w4.

A total of 2875 unique CRISPR spacers were extracted from the five wound microbiomes. Only 625 (22%) of these spacers found match protospacers in the MGE database. The bacteria-phage network (**Figure 5**; right) shows that the most phage sharing is found between w3 and w5, which is consistent with the taxonomic similarity between these microbiome datasets. Microbiome w4 has a very different (and also the simplest) taxonomic composition with *P. aeruginosa* contributing more than 98% of the total reads in this microbiome dataset, and only two phages were identified for this microbiome, which is not surprising as this microbiome has low composition complexity with only one dominating species. Finally, although wound microbiome w1 and w2 have similar bacterial composition, they share few common MGEs. This could be explained by that the dominating species in these two microbiome is *S. aureus*, which is a species that rarely contains CRISPR-Cas systems in its genomes. We observed that among 12 thousands of *S. aureus* isolates, only 0.55% of them contain CRISPR–Cas systems (Mortensen et al., 2021).

## Discussion

CRISPR-Cas systems are themselves subject to horizontal transfer (Singh et al., 2021). We reason that Type V-A CRISPR-Cas system found in the *P. vulgatus* pangenome analyzed was likely acquired through horizontal gene transfer since the occurrence was rare (only found in one isolate). Since no protospacers were found that match the Type V-A spacers, the Type V-A spacers did not cause false identification of phages that interact with this bacterial species. However, it is possible that the mapping of spacers found in CRISPR arrays and segments in phage genomes could lead to false prediction of bacteria-phage interactions. This is a potential limitation of our approach, and any approach that uses spacers for phage host prediction.

The three collections of genomic/metagenomic datasets received different ratios of spacers that have matches in phages. The *P. vulgatus* pangenome has the highest ratio (about 78% of identified spacers have their counterparts in phages/pladmids). This result is expected since *P. vulgatus* is a bacterial species that is commonly found in the gut microbiome, and metagenomic sequencing projects have resulted in the accumulation of phages that are associated with this species. For comparison, the human gut MAG collection has a lower ratio (45%), indicating that using the existing MGE database might still be insufficient for comprehensive identification of phage-bacteria interaction in gut microbiome, which has been shown to be highly variable between individuals, and different time points of the same individuals (Zaoli and Grilli, 2021). The wound microbiome has the lowest ratio of its spacers matched to phages (22%), reflecting that the MGE database is underrepresented for phages that invade species in the wound microbiome.

We showed that results from network-based analyses can provide insight into the interaction between phages and bacteria (such as the differential defense activities of the CRISPR-Cas against different phages), and the modularity of the networks can be utilized for prediction of phage hosts. For example, module 9 in the gut bacteria-MGE network (**Figure 4C**) is likely a result of the specific interaction between Lachnospiracea and its invaders, and therefore can be used to provide confident prediction of hosts for the phages with unknown hosts.

It was found that about half of bacterial genomes contain CRISPR-Cas systems, while most archaea contain them (Koonin et al., 2017; Zhang and Ye, 2017). Archaea are rare in human gut microbiome, as a result, using our pipeline can reveal the potential invaders of about half of the microbial species in the gut microbiome (see Results). An apparent limitation of our pipeline is that it won’t be able to reveal the invaders of the genomes that don’t contain CRISPR-Cas systems. Nevertheless, our work helped reveal the bacteria-MGE interactions that are mediated through the CRISPR-Cas systems, one of the most important defense systems that microbial organisms have to fight against their invaders. Finally, we applied a greedy algorithm to select non-redundant set of identified MGEs from the pipeline, which was to simplify the interaction networks. This step would eliminate some bacteria-MGE interactions that users might be interested in. Users could look back into the intermediate outputs from the pipeline to recover those interactions if needed.

## Data availability statement

The original contributions presented in the study are included in the article/supplementary material. Further inquiries can be directed to the corresponding authors.

## Author Contributions

MM carried out the implementation of some steps of the pipeline, participated in analysis (the human gut microbiome) and draft of the manuscript. SZ participated in the analysis (the wound microbiome) and writing of the manuscript. KM participated in the analysis and writing of the manuscript. YY conceived the study, participated in its design and implementation, participated in the analysis, and helped to draft the manuscript. All authors contributed to the article and approved the submitted version.

## Funding

This study was supported by the NIH grant R01AI143254 and NSF grant EF-2025451.

## Conflict of interest

The authors declare that the research was conducted in the absence of any commercial or financial relationships that could be construed as a potential conflict of interest.

## Publisher’s note

All claims expressed in this article are solely those of the authors and do not necessarily represent those of their affiliated organizations, or those of the publisher, the editors and the reviewers. Any product that may be evaluated in this article, or claim that may be made by its manufacturer, is not guaranteed or endorsed by the publisher.
